# Cortical synchrony is reduced in Alzheimer's disease and relates to arousal state

**DOI:** 10.1002/alz.71547

**Published:** 2026-06-11

**Authors:** Michael C. B. David, Emma‐Jane Mallas, Magdalena A. Kolanko, Martina Del Giovane, Danielle L. Kurtin, Ramin Nilforooshan, Karl A. Zimmerman, Cristina Bonet Olivares, Peter J. Lally, CR&T Group of UKDRI, David J. Sharp, Paresh A. Malhotra, Gregory Scott

**Affiliations:** ^1^ UK Dementia Research Institute Care Research and Technology Centre Imperial College London London UK; ^2^ Imperial College London, Brain Sciences London UK; ^3^ Division of Psychiatry Imperial College London London UK; ^4^ Surrey and Borders Partnership NHS Foundation Trust Leatherhead UK

**Keywords:** Alzheimer's disease, arousal, locus coeruleus, cognition, fMRI, pupillometry, LEiDA, synchrony, metastability, criticality

## Abstract

**INTRODUCTION:**

The brain is a complex dynamical system, influenced by arousal state. Cortical synchrony supports information processing and is disrupted in Alzheimer's disease (AD). Locus coeruleus (LC) integrity and pupillometry index arousal system structure and function.

**METHODS:**

Sixty‐four AD and 26 controls underwent resting‐state pupillometry‐fMRI. Neuromelanin MRI and Addenbrooke's Cognitive Examination were conducted. Mean and standard deviation of blood oxygen level dependent (BOLD) phase coherence yielded synchrony and metastability, respectively. Leading Eigenvector Dynamics Analysis (LEiDA) produced coherence‐based states.

**RESULTS:**

AD had reduced global synchrony [*b *= −0.90, *p* < 0.001], metastability [*b *= −0.61, *p* < 0.01], LEiDA “global coherence state” occupancy [*b *= −0.06, *p* < 0.01], and LC integrity [*b *= −0.37, *p* = 0.01]. Synchrony [*b *= 0.19, *p* = 0.01] and LC integrity [*b *= 0.17, *p* < 0.01] related to cognition and one another [*b *= 0.27, *p* = 0.01]. Pupil‐linked arousal correlated with synchrony and global coherence state maintenance.

**DISCUSSION:**

In health, cortical activity shows widespread but dynamic synchrony across regions to meet changing demands. In AD, arousal dysfunction appears to disrupt these dynamics, impacting cognition.

## Highlights

- Pupillometry‐functional magnetic resonance imaging (fMRI), locus coeruleus MRI, and cognitive testing were conducted in Alzheimer's disease (AD)
- AD showed reduced synchrony and maintenance of the Leading Eigenvector Dynamics Analysis (LEiDA) “global coherence state”
- Metastability (variability in cortical synchrony over time) was also reduced in AD
- Global synchrony related to arousal state and different aspects of cognition
- Arousal dysfunction may explain disruption of cortical dynamics and cognition in AD

## 1 BACKGROUND

Cortical dynamics, that is, fluctuations in integrated brain activity, play a key role in cognition.^1^, ^2^ Alzheimer's disease (AD) disrupts these dynamics.^3^, ^4^, ^5^ Recent animal and human work demonstrates that fluctuations in brain activity are largely explained by arousal state,^6^, ^7^ which may act as a brain‐wide control mechanism of relevance for understanding neurological disease.^7^ We previously reported a strong relationship between arousal state (pupil size) and cortical dynamics (electroencephalography [EEG]) that is preserved in AD.^8^ We extend these findings, this time combining functional magnetic resonance imaging (fMRI) with pupillometry and neuromelanin MRI of the locus coeruleus (LC), to further examine how arousal dysfunction relates to cortical dynamics and cognitive function in AD.

Synchrony is one measure of cortical dynamics that may be particularly relevant to cognitive impairment and dementia. Across spatial scales, neuronal synchrony is linked to attention, memory, and language function.^9^, ^10^ The phase alignment of neuronal activity observed with non‐invasive (EEG/magnetoencephalography [MEG]/fMRI), or invasive (field/spike) recordings provides measures of synchrony required for information processing that supports cognition (see ^11^, ^12^ for reviews). We have previously shown how synchrony measured using fMRI blood oxygen level dependent (BOLD) varies with attentional state,^13^ whilst others have reported changes in synchrony across cognitive disorders, including Parkinson's disease, schizophrenia, and AD.^12^, ^14^

The mechanisms underlying altered cortical dynamics in AD are unclear. One potential explanation is that they reflect changes in arousal function.^15^ Neurodegenerative pathology in brainstem nuclei^8^, ^16^ represents a plausible cause of AD‐related changes in cortical dynamics.^17^, ^18^, ^19^ In particular, tau accumulation followed by cell loss occurs in the pontine LC, the primary source of central noradrenaline and a key nucleus in arousal control, early in the disease process.^20^ MRI sequences designed to produce contrast in neuromelanin‐containing cells in the LC can be used to measure its structural integrity.^21^

Pupil size provides a functional measure of arousal state, showing close coupling with LC‐noradrenergic activity in mice,^22^ monkeys,^23^ and humans.^24^ Using pupillometry to track LC‐driven arousal alongside simultaneous measures of cortical activity has helped understand how arousal fluctuations influence brain state dynamically.^6^, ^25^, ^26^ In fact, it is possible to accurately reconstruct large‐scale dynamics in the mouse cortex, measured using a range of imaging modalities, from pupil size alone.^7^ Pupillometry has also revealed arousal dysfunction in AD, including attenuated dilation responses to “oddball” stimuli.^8^ Simultaneous pupillometry‐fMRI is a particularly powerful means of measuring the influence of arousal state on cortical activity since arousal fluctuates relatively slowly, so the effects can still be captured despite the low temporal resolution of fMRI.^6^ This approach has been used in health although not reported in AD.^1^, ^26^, ^27^, ^28^

Decreases in fMRI BOLD‐derived synchrony between specific networks have been shown in AD.^5^ However, recent work has highlighted that the influence of arousal on cortical activity occurs on a global scale.^6^, ^7^ This motivated us to quantify synchrony as a single metric calculated across the entire cortex to capture activity likely to reflect the influence of arousal as a system‐level controller. In addition to synchrony calculated as mean phase coherence,^29^ the standard deviation gives a measure of metastability – a marker of the brain's ability to flexibly switch between different patterns of activity.^2^ Metastability has been shown to be reduced in cognitive disorders including AD.^4^, ^5^, ^30^, ^31^ We also used Leading Eigenvector Dynamics Analysis (LEiDA) to capture the global connectivity pattern (states) volume‐by‐volume.^32^ Extending previous AD work, we related these synchrony‐based markers of cortical activity to arousal state.^5^, ^33^, ^34^

In addition to combined pupillometry‐fMRI we obtained neuromelanin MRI. This set of modalities, to our knowledge never combined in clinical AD, enabled us to assess the impact of arousal dysfunction on synchrony‐based measures of cortical dynamics and cognitive function. We tested five main hypotheses: (1) Synchrony, metastability, and time spent in the LEiDA global coherence state are reduced in AD. (2) Reductions in synchrony relate to impaired cognition. (3) Reduced LC integrity relates to lower synchrony. (4) Higher pupil‐linked arousal is associated with greater synchrony and maintenance of the LEiDA “global coherence state”. (5) The pupil‐brain relationship is preserved in AD.

**RESEARCH IN CONTEXT**

1. **Systematic review**: We searched PubMed and Google Scholar for studies on cortical dynamics, arousal, and locus coeruleus (LC) integrity in Alzheimer's disease (AD). Prior work shows LC degeneration occurs early in AD and influences cognition. However, few studies link LC integrity or pupillometry‐based arousal measures to large‐scale brain dynamics in AD.
2. **Interpretation**: Our findings demonstrate that AD is associated with impaired synchrony and flexibility of cortical brain activity. LC integrity and pupil‐linked arousal significantly relate to these dynamics and cognitive performance. This suggests that arousal dysfunction plays a key role in the disruption of cortical dynamics and cognition.
3. **Future directions**: How disruptions in arousal‐related cortical activity directly impact behaviour in AD, for example, during cognitive tasks, should be investigated. Also, experimental medicine studies should explore whether arousal‐modulating interventions can restore cortical synchrony and metastability to improve cognition.

## 2 METHODS

### 2.1 Participants

AD participants were recruited as part of both the Minder and Physiological Correlates of Noradrenergic Add‐on Therapy (PCNorAD) studies. Minder is an ongoing longitudinal community‐based cohort study of people with dementia run by the Care Research and Technology Centre of the UK Dementia Research Institute.^35^ PCNorAD is an experimental medicine add‐on study investigating noradrenergic dysfunction in patients with AD who completed the NorAD clinical trial^36^ at least one month previously. Healthy older controls (HC) were also recruited as part of PCNorAD.

AD participants had a pre‐existing clinical diagnosis of AD but were also discussed within a multi‐disciplinary team (MDT) meeting, comprised of neurologists, psychiatrists and neuroradiologists, as part of this study, to confirm the diagnosis. Research and clinical neuroimaging and cognitive assessments plus clinical history and AD biomarkers, where available, were considered. In the AD group, amyloid positivity was confirmed on positron emission tomography (PET) *N* = 14, PET and cerebrospinal fluid (CSF) *N* = 2, or CSF only *N* = 1, whilst two potential AD participants were not included due to negative PET/CSF. Also, a subset of participants (32 AD and 23 HC) had plasma phosphorylated tau protein 217 (pTau‐217) measured as part of the wider PCNorAD and Minder study protocol, the results of which were also used by the MDT to confirm the diagnosis. In total, amyloid biomarkers were available for 35/64 AD and 23/26 HC. See section 2.6 for details of plasma pTau‐217 testing and Table 1 for group comparison statistics. On the day of study visits, participants were asked to not consume caffeine, and drugs with noradrenergic action were withheld. HC were eligible if > 60 years old and with no symptoms of cognitive impairment. All participants were free of major neurological or psychiatric illnesses besides AD.

**TABLE 1:** Participant characteristics.

	HC	AD	Statistic	*p*‐value
Total included in rsfMRI analysis, N	26	64		
Pupillometry, N	24	55		
Neuromelanin, N	26	54		
DTI, N	25	52		
Cognitive testing (ACE), N	26	58		
Age, years, mean (SD)	76.3 (5.5)	74.6 (8.5)	*t* = −1.12	0.27
Sex, % female	46.2	57.8	*χ* ^2^ = 0.71	0.40
ACE, mean (SD)	96.2 (3.0)	59.4 (17.2)	*t* = −15.77	< 0.001
Attention	17.6 (0.7)	10.8 (4.0)	*W* = 77.5	< 0.001
Fluency	12.5 (1.4)	6.9 (3.2)	*W* = 73.5	< 0.001
Language	25.7 (0.5)	20.1 (5.0)	*W* = 141	< 0.001
Memory	24.8 (1.6)	10.1 (5.3)	*W* = 24	< 0.001
Visuospatial	15.7 (0.6)	11.6 (4.4)	*W* = 204	< 0.001
Full‐time education, years, mean (SD)	15.4 (3.8)	13.8 (3.0)	W = 627.5	0.07
AChEI, drug, daily dose (n)		Don 5 mg (7), Don 10 mg (31), Gal 16 mg (4), Gal 24 mg (1), Riv 1.5 mg (1), Riv 6 mg (1), Riv 9.5 mg (3), nil (16)		
Dementia symptoms, years, mean (SD)		5.2 (3.0)		
pTau‐217, mean (SD)	0.4 (0.2)	1.0 (0.5)	W = 774	< 0.001

*Note*: The ACE was conducted on the day of the rsfMRI. For nine subjects, DTI data were taken from an alternative scan visit to that of the rsfMRI. pTau‐217 measured within 1 year of the rsfMRI in 32 AD and 23 HC. *T*‐test or Wilcoxon test used to compare groups for continuous variables, based on normality; chi‐square for % female.

Abbreviations: ACE, Addenbrooke's Cognitive Examination – III; AChEI, acetylcholinesterase inhibitors; AD, Alzheimer's disease; Don, donepezil; DTI, diffusion tensor imaging; Gal, galantamine; HC, healthy control; MRI, magnetic resonance imaging; pTau‐217, phosphorylated tau protein 217; Riv, rivastigmine; rsfMRI, resting‐state functional MRI.; SD, standard deviation.

Addenbrookes Cognitive Examination‐III (ACE)^37^ was performed on the day of the MRI scan. ACE scores were used for participant‐level analysis of MRI metrics in relation to cognition. HC were to be excluded from the study if there was evidence of objective cognitive impairment defined as an ACE of < 88.

### 2.2 Ethical approval

The Minder and PCNorAD studies were approved by the Health Research Authority's London‐Surrey Borders Research Ethics Committee (19/LO/0102) and London‐Central Research Ethics Committee (18/LO/0249), respectively. All participants with capacity to consent provided written informed consent for participation and for their data to be published. Those without capacity were enrolled on recommendation of an assigned consultee.

### 2.3 MRI acquisition

Data were acquired using a Siemens Verio 3T MRI scanner with a 32‐channel head coil. Participants underwent a T1‐weighted three‐dimensional magnetisation‐prepared rapid acquisition gradient‐echo (3D‐MPRAGE) sequence. The parameters were: flip angle = 9°, echo time (TE)  = 2.98 ms, repitition time (TR)  = 2300 ms, inversion time = 900 ms, bandwidth = 240 Hz/pixel, acquisition matrix = 256 × 240 × 160, voxel size = 1.0 × 1.0 × 1.0 mm, GRAPPA (GeneRalized Autocalibrating Partially Parallel Acquisitions) acceleration factor = 2. Acquisition time was 05 min 03s.

LC contrast data were acquired using the following 3D T2*‐weighted multi‐echo gradient‐echo with magnetisation transfer preparation pulse sequence which was aligned perpendicularly to the plane of the participant's brainstem based on the sagittal reconstruction from the MPRAGE sequence and used the following parameters: flip angle = 20°, TE = 7.5,15.0,22.5 ms, TR = 62 ms, acquisition matrix = 384 × 384 × 48, voxel size = 0.67 × 0.67 × 1.34 mm, bandwidth = 230 Hz/pixel, GRAPPA acceleration factor = 2, slice partial Fourier factor = 6/8. Acquisition time was 06 min 32s. The first echo time was analysed.

Diffusion tensor imaging (DTI) data were acquired using a spin‐echo echo‐planar imaging sequence with the following parameters: TR = 9500 ms, TE = 103 ms, voxel size = 2.0 × 2.0 × 2.0 mm, and 64 axial slices with no interslice gap, GRAPPA acceleration factor = 2, base resolution = 128, partial Fourier = 6/8 was used in the phase encoding direction and bandwidth = 1562 Hz/pixel. The diffusion acquisition included two b‐values: *b* = 0 and *b* = 1000s/mm^2^, with 3 and 64 diffusion directions acquired at each, respectively. Acquisition time was 11 min 15s.

Ten minutes of resting‐state fMRI data were obtained using a T2*‐weighted gradient‐echo echo planar imaging (EPI) sequence^38^ with whole‐brain coverage and the following parameters: TE  = 32.2 ms, TR = 2000 ms, 54 interleaved ascending slices with thickness 2.6 mm, no interslice gap, in‐plane resolution 2.6 × 2.6 mm, flip angle 80°, field of view 21 × 21 cm, matrix 80 × 80, multi‐band acceleration factor 2. Participants were asked to keep their eyes open, stay awake and stare at a central fixation cross but let their mind wander and think of nothing in particular. Lighting was kept constant.

MRI scans were acquired once for PCNorAD participants and approximately annually for Minder participants for as long as they remained in the study. In total, 161 scan visits were conducted, including 123 participants (94 AD, 29 HC). One HC was subsequently excluded on the basis of a low ACE score of 85, 24 AD scan visits and one HC visit were excluded as the resting‐state sequence was not completed, and one AD visit's fMRI data was excluded due to acquisition artefact. Where a participant completed multiple usable fMRI visits, only one was selected for pre‐processing based on the availability of contemporaneous usable pupillometry, ACE, and neuromelanin data, in that order of priority. This resulted in fMRI data from 109 participants being pre‐processed, 19 of which were then excluded based on their framewise displacement (FD) (see Supplementary Methods), and so ultimately 90 were included in the analysis (64 AD, 26 HC).

### 2.4 MRI pre‐processing and analysis

#### 2.4.1 Structural data

T1 images underwent brain extraction using HD‐BET^39^ and gray matter segmentation using FMRIB's Automated Segmentation Tool^40^ to give total gray matter volume and estimated total intracranial volume. LC contrast was quantified using the method detailed in David *et al.* ^8^ Briefly, the LC was manually identified by two independent raters blind to study group. Six did not complete the neuromelanin sequence and a further four participants’ neuromelanin scans were not analysed due to acquisition/movement artefact (all AD). For those deemed analysable, a 5‐voxel region centred at the voxel with the highest contrast in the putative LC region, on both the left and right, was defined. The contrast ratio between the LC and a 7 × 7 voxel square region in the nearby pons was calculated using the following equation:

(mean of left and right LC contrast – mean reference contrast)/standard deviation of reference contrast.^41^

This was conducted across five consecutive slices and then averaged to give a single value. This was then averaged between raters who had a high intraclass correlation coefficient of 0.88–0.96. Where there was a large disagreement between raters (contrast ratio > 0.45), scans were re‐examined, and a consensus was reached.

DTI offers a quantitative approach for examining white matter structure in vivo, with fractional anisotropy (FA) providing a measure of white matter integrity.^42^ DTI data were processed using FMRIB's Software Library (FSL)'s diffusion toolbox following established procedures (see Supplementary Methods).^43^ The mean FA across the entire skeleton was calculated for each participant as a measure of overall white matter integrity.

#### 2.4.2 Functional data

Functional imaging pre‐processing was performed using FEAT, in FSL, according to standard procedures (see Supplementary Methods).^44^ In terms of correcting for movement artefact, first, FD, a measure of head movement from one frame to the next,^45^ was obtained for each volume using the FSL motion outliers tool. Although previous work in AD has not chosen to exclude participants based on mean FD,^46^, ^47^, ^48^ we took a more stringent approach and excluded scan visits with a mean FD > 0.55.^49^ Of the acquisitions selected for pre‐processing, 18/82 (22%) AD and 1/27 (4%) HC participants were excluded on FD. In those kept for analysis, there was no group difference in FD [*p* > 0.05], and the final group‐level mean FD [*HC* = 0.29; *AD* = 0.31] was comparable to other work.^48^

**FIGURE 1:**
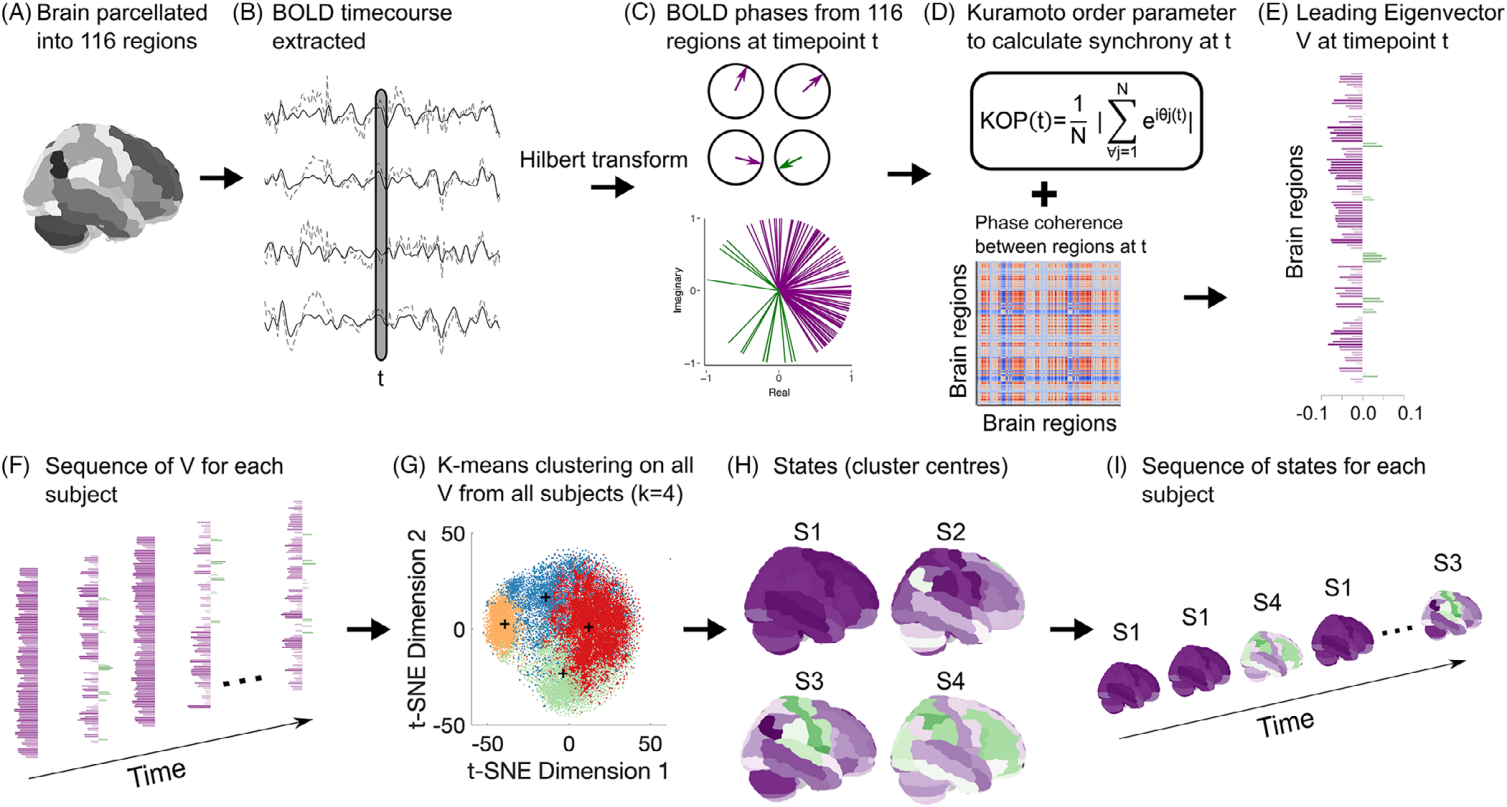
fMRI LEiDA analysis. (A–B) BOLD activity from 116 regions filtered between 0.02–0.1 Hz. (C) Each region's time course was Hilbert transformed to yield the phase at each timepoint/volume (t). (D) At each t, the phase coherence between all pairs of regions was measured to give synchrony – the mean and standard deviation of which across the whole acquisition was used to give the overall synchrony and metastability value for each subject. The phase coherence between regions was also used to produce unique matrices for every volume, for use in the LEiDA analysis. (E–F) The leading eigenvector (V) was extracted from each matrix to give a sequence of unique V. (G–H) K‐means clustering was applied to all V from all subjects to produce cluster centres, taken as ‘states’. (I) States were back‐projected onto V to give a state sequence for each subject. Dummy data used for representation of the methods. BOLD, blood oxygen level dependent; fMRI, functional magnetic resonance imaging; LEiDA, Leading Eigenvector Dynamics Analysis.

Following data cleaning, a timecourse of averaged activity was extracted from voxels belonging to each of the 116 ‘AAL Atlas 2’ regions, covering the whole brain.^50^ The use of the AAL2 atlas that includes the cerebellum is commensurate with our previous LEiDA work,^51^ and that of multiple other groups.^52^, ^53^ This previous work has shown patterns of connectivity involving the cerebellum that are specific to certain LEiDA states and hencewe felt,a priori, it was justified to use this atlas to define the states. The cerebellum is increasingly recognised as important in the control of arousal state and cognitive functions^54^ and exhibits specific functional connectivity alterations in AD.^55^ Including cerebellar nodes allowed a more complete representation of large‐scale network dynamics. Each regional timecourse of activity, for each subject independently, was demeaned and band‐pass filtered by a second‐order Butterworth filter (0.02–0.1 Hz).^29^ Timecourses were Hilbert transformed. The first and last volume from the phase timecourse were removed, and then the Kuramoto order parameter (KOP)^56^ was computed as a measure of synchrony between regions (Figure 1). The KOP ranges from 0 (fully unsynchronised) to 1 (fully synchronised) and is defined in the equation below, where *θ*jθj(t) is the signal phase of an oscillator (region) j at a given time.

$$\mathrm{KOP}\left(t\right)=\frac{1}{N}\left|\sum_{j=1}^{N}e^{i\theta j(t)}\right|$$

Once the KOP was obtained for each volume, the mean and standard deviation over time was calculated for each participant to give the overall global synchrony and metastability, respectively.^31^, ^57^

Brain states were then defined using the LEiDA approach.^32^ Again, after removing the first and last volume, an instantaneous functional connectivity matrix between the phases of 116 regions was computed by the following equation:

FC(n,p,t)=cos(θ(n,t)−θ(p,t))

where FC (*n*, *p*, *t*) is a functional connectivity matrix containing the BOLD phase coherence between brain areas *n* and *p* at time *t*.

Then the leading eigenvector was extracted from the functional connectivity matrix, rather than considering the connectivity between all regions (the whole upper triangle of a matrix). This captures the dominant phase coherence connectivity pattern at time *t*, reducing the dimensionality from N(N−1)/2 to N (where N is the number of regions), while still accounting for over 50% of the variance.^32^ For each volume, the associated leading eigenvector separates elements with different signs (positive or negative), dividing the regions into two communities.^58^ The leading eigenvectors from all volumes from all participants were subsequently clustered using *k*‐means with a range of 2–10 chosen as *k* values. The centre of each cluster represents a distinct connectivity state, and each volume of the timeseries was assigned to one of these clusters, producing a state timeseries (Figure 1). An optimum value for *k* of 4 was established based on the Silhouette score (Figure S1).^59^, ^60^, ^61^, ^62^, ^63^

The probability of occurrence, dwell time, and number of visits for each state was calculated for each participant. The probability of a state is the proportion of volumes across the whole acquisition in which the participant is in that state. The dwell time is the average number of consecutive volumes spent in a given state for each visit to that state.^32^ For each participant, a 4 × 4 transition matrix was constructed, representing the probabilities of transitioning from State 1–4 to each of the other three states, or remaining in the current state. The four possible transitions from each origin state (including remaining in the current state) were represented as a proportion of all transitions from that origin state (matrix row). The transition matrices were then averaged across participants within each group to produce group‐level matrices.

### 2.5 Pupillometry

Pupillometry data were pre‐processed according to established in‐house procedures^8^ before being quality checked. The pupil area (referred to here as size) was measured using an EyeLink 1000 MRI‐compatible Long‐Range Mount eye‐tracker (SR Research Ltd., Canada) from the right eye for all participants. A five‐point calibration procedure was conducted prior to the resting‐state to ensure accurate gaze‐position tracking. Data were recorded at 1000 Hz and pre‐processed including (in order) blink interpolation, filtering, z‐scoring, and downsampling to 0.5 Hz. A low‐pass filter at 2 Hz was used to remove high‐frequency noise but preserve low frequency pupil fluctuations as these have been shown to correlate most strongly with the BOLD signal.^64^ As in David *et al.*,^8^ following pre‐processing, data were inspected for quality and excluded if excessively artefactual or incomplete (10 AD and one HC). The included pupillometry timecourses were then shifted by two volumes (4 s) forward in time relative to the fMRI data. The hemodynamic response function dictates that the BOLD signal lags behind neuronal activity by ∼5s,^65^ whilst pupil size appears to strongly correlate with cortical activity at a lag of ∼1s.^8^, ^25^ Therefore, to align the pupils with the BOLD signal to measure the same underlying phenomena, a shift of ∼4s is required.

The correlation between the pupillometry timecourse and the instantaneous synchrony timecourse was then calculated for each participant. A non‐zero correlation coefficient was tested for across all participants and the strength of the correlations was compared between groups using a *t*‐test.

To further explore the relationship between pupil size and synchrony, each volume was assigned to one of ten bins according to the pupil size within that volume.^8^ Each bin was equally spaced along the range of pupil size values for that participant. The corresponding synchrony value for all the volumes in each bin was averaged giving a single synchrony value per bin, per participant. These values were then averaged across participants to compute a group‐level synchrony value for each bin. Then, to explore how pupil size differed as a function of LEiDA state, the average pupil size was compared for volumes in each of the four states and for volumes either side of state transitions.

### 2.6 Plasma pTau‐217

For a subset of 32 AD and 23 HC, blood samples were collected using ethylenediaminetetraacetic acid‐coated tubes. After 30 to 120 minutes at room temperature, samples were centrifuged at 2500 g at 4°C for 20 min and frozen at −80°C. Plasma pTau‐217 was quantified using the ALZpath pTau217 assay (Quanterix, Inc.).

### 2.7 Statistical analysis

Linear regression models were used to compare groups for LC contrast, mean FA, gray matter volume, ACE, synchrony, and metastability. When testing for group differences, age and sex were used as covariates in all models, as was years of education for models testing cognition and estimated total intracranial volume for models testing total gray matter volume. When testing for relationships between two non‐categorical variables across all participants, group and years of dementia symptoms were additionally included as a covariate to account for overall disease stage.

We compared the transition behaviours in relation to the LEiDA states between groups, including the probabilities of moving from any one state to another as well as the total number of transitions. Additionally, the following metrics were calculated for each state (see functional data analysis section): probability, number of visits, mean dwell time, and synchrony during that state. The effect of group and state (as well as their interaction) were then tested using a linear mixed effects model including a random intercept for each participant, with the following equation:

$$MetricValue∼Group*State+\left(1|Participant\right)$$

An analysis of variance (ANOVA) was then performed on the fitted model to assess main and interaction effects. Post‐hoc comparisons of group differences were performed within each state using estimated marginal means, and pairwise contrasts were computed. These post‐hoc p‐values are reported following false discovery rate (FDR) correction. Linear mixed effects models were applied across all volumes, with participant as a repeated measures term to compare pupil size during State 1 versus not State 1, and preceding state transitions versus remaining in the same state. All analyses were conducted using RStudio Version 2024.04.2 + 764 (Posit, PBC) and MATLAB R2021a (MathWorks) including the ‘lmer’ function from the lme4 package and the ‘emmeans’ function from the emmeans package.

## 3 RESULTS

### 3.1 Participant characteristics

Sixty‐four AD participants and 26 HC underwent a 10‐minute resting‐state fMRI scan with simultaneous pupillometry, as well as neuromelanin MRI, diffusion MRI, and cognitive testing (see Table 1 for numbers included for each analysis after exclusions). Groups were matched for age, sex, and years of education. Forty‐eight AD were on acetylcholinesterase inhibitors at the time of testing. The average length of dementia symptoms in the AD group was 5.2 ± 3.0 years (mean ± SD).

### 3.2 Synchrony is reduced in AD and relates to LC integrity

**FIGURE 2:**
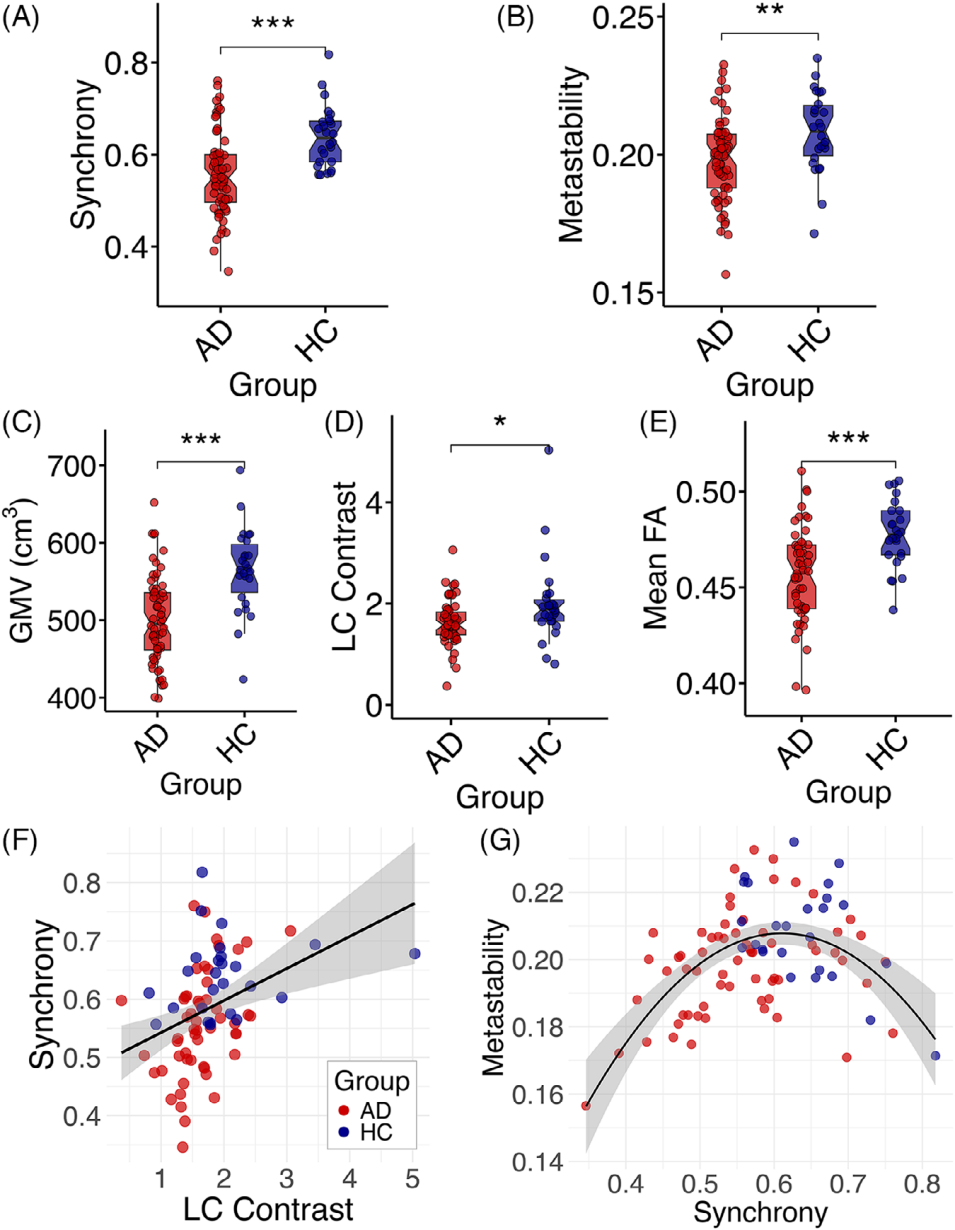
Structural and functional imaging measures. (A–E) Box plots comparing groups on structural and functional imaging measures. Significance indicators are the result of regression models accounting for covariates. (F) Scatter plot showing significant relationship between LC contrast and synchrony. For illustrative purposes, linear best fit were plotted through raw data from all participants. Statistical results reported in text include group as covariate. LC contrast calculated as a ratio relative to the central pons. (G) Scatter plot showing a significant quadratic relationship between synchrony and metastability. A single quadratic line is fitted to data from all participants. Shaded error bars show standard error of the mean. *p < 0.05; **p < 0.01; ***p < 0.001. AD, Alzheimer's disease (red); FA, fractional anisotropy; GMV, gray matter volume; HC, healthy control (blue); LC, locus coeruleus.

We first tested the hypothesis that synchrony would be reduced in AD and relate to the integrity of the LC, as a key arousal nucleus. Synchrony [*b *= −0.90, SE = 0.22, *t*(86) = −4.17, *p* < 0.001] and metastability [*b *= −0.61, SE = 0.22, *t*(86) = −2.72, *p* < 0.01] over 10‐mins of resting‐state (Figure 2A/B) were significantly lower in AD. Also reduced in AD were total gray matter volume [*b *= −0.57, SE = 0.09, *t*(85) = −6.01, *p* < 0.001], LC integrity, measured as the contrast ratio on neuromelanin MRI [*b *= −0.60, SE = 0.24, *t*(76) = −2.54, *p* = 0.01], and mean FA [*b *= −0.84, SE = 0.21, *t*(73) = −4.05, *p* < 0.001], a measure of white matter integrity (Figure 2C/D/E).

Across all participants, adjusting for covariates including group, the relationship between synchrony and metastability was best captured by a quadratic curve, with metastability peaking at intermediate synchrony [*b *= −0.72, SE = 0.12, *t*(83) = −6.08, *p* < 0.001] (Figure 2G). The quadratic model showed a lower Akaike information criterion than a linear model (−528 vs −497). There was a significant positive relationship between LC contrast and synchrony [*b *= 0.27, SE = 0.10, *t*(74) = 2.61, *p* = 0.01] (Figure 2F), but no group interaction [*p* = 0.07]. There was no relationship between LC and metastability [*p* = 0.26]. Gray matter volume and FA did not relate to synchrony nor metastability [all *p* > 0.05]. Aggregated across compounds and dosages, acetylcholinesterase inhibitors use had no effect on LC contrast, metastability, or synchrony [all *p* > 0.05, *N* = 48 on drug vs *N* = 16 not]. We were underpowered to investigate the specific effects of the different cholinergic compounds.

### 3.3 Synchrony, LC, and white matter integrity independently relate to cognition

**FIGURE 3:**
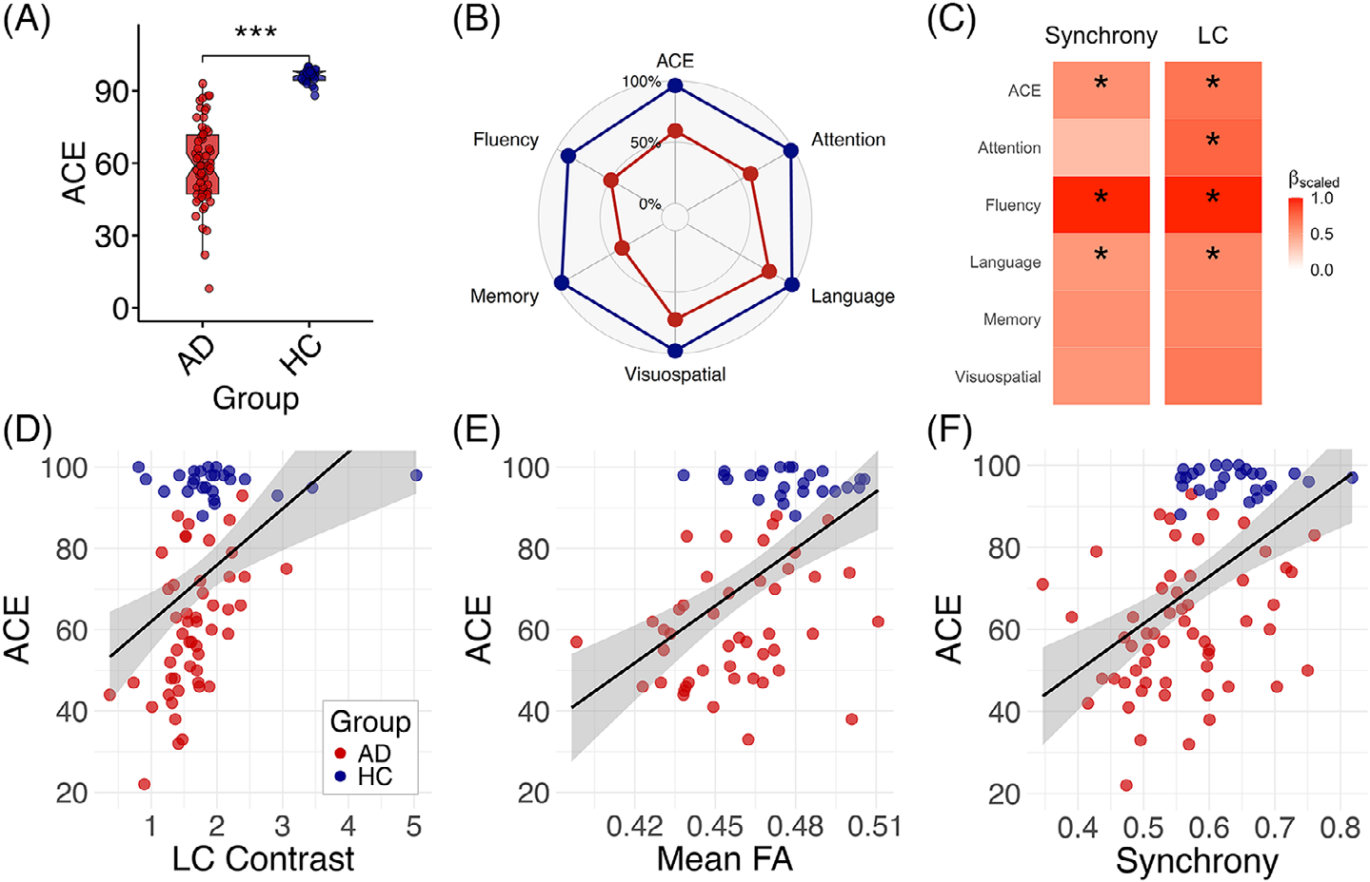
Imaging measures and cognition. (A) Box plots comparing groups on ACE total score. Significance indicator is the result of regression models accounting for covariates. ***p < 0.001. (B) Radar plot showing the scores in the ACE, overall and by sub‐domain, rescaled for comparison between domains and, split by group. (C) Heatmap showing the results from linear models for all subjects, accounting for group, age, sex, and education, testing the relationship between both synchrony and LC contrast with the ACE total and sub‐domain scores. Each combination of cognitive outcome measure and imaging predictor variable run as separate models, without correction for multiple comparisons. The colour represents the β coefficient from the model, rescaled by dividing by the average score for that cognitive measure. The asterisks represent significant relationships (*p* < 0.05). (D–F) Scatter plots showing significant relationship between imaging measures and total ACE score. For illustrative purposes, linear best fit was plotted through raw data from all participants. Statistical results reported in text include group as covariate. ACE, Addenbrooke's Cognitive Examination – III; AD, Alzheimer's disease (red); FA, fractional anisotropy; HC, healthy control (blue); LC, locus coeruleus.

Across all participants, accounting for group, both synchrony [*b *= 0.19, SE = 0.07, *t*(77) = 2.54, *p* = 0.01] and LC contrast [*b *= 0.17, SE = 0.06, *t*(69) = 2.69, *p* < 0.01] were positively related to total ACE (Figure 3D/E) whereas metastability was not. Regarding the relationship between LC and ACE, there was a significant group interaction. In the AD group, the LC was strongly positively related to ACE [*b *= 0.50, SE = 0.11, *t*(44) = 4.46, *p* < 0.001] whereas in the HC group there was no relationship. There was no group interaction for the relationship between synchrony and ACE. When examining the sub‐domain scores of the ACE, LC was positively related to attention [*b *= 0.17, SE = 0.08, *t*(69) = 2.14, *p* = 0.04], fluency [*b *= 0.18, SE = 0.08, *t*(69) = 2.11, *p* = 0.04], and language [*b *= 0.21, SE = 0.09, *t*(69) = 2.42, *p* = 0.02], with no relation to other sub‐domains. Synchrony was positively related to fluency [*b *= 0.23, SE = 0.09, *t*(77) = 2.67, *p* < 0.01] and language [*b *= 0.25, SE = 0.10, *t*(77) = 2.47, *p* = 0.02] only (Figure 3C, Table S1).

To then test for the significance of synchrony in relation to cognition, independent of recognised AD‐related structural abnormality, we controlled for imaging measures of neurodegeneration in the white and gray matter (mean FA, gray matter volume). When all were included as covariates in the same model, synchrony [*b *= 0.16, SE = 0.07, *t*(61) = 2.27, *p* = 0.03], FA [*b *= 0.18, SE = 0.07, *t*(61) = 2.69, *p* < 0.01], and gray matter volume [*b *= 0.70, SE = 0.16, *t*(61) = 4.36, *p* < 0.001] all independently related to cognitive function measured by the ACE.

### 3.4 A ‘global coherence state’ predominates across all participants when using LEiDA

**FIGURE 4:**
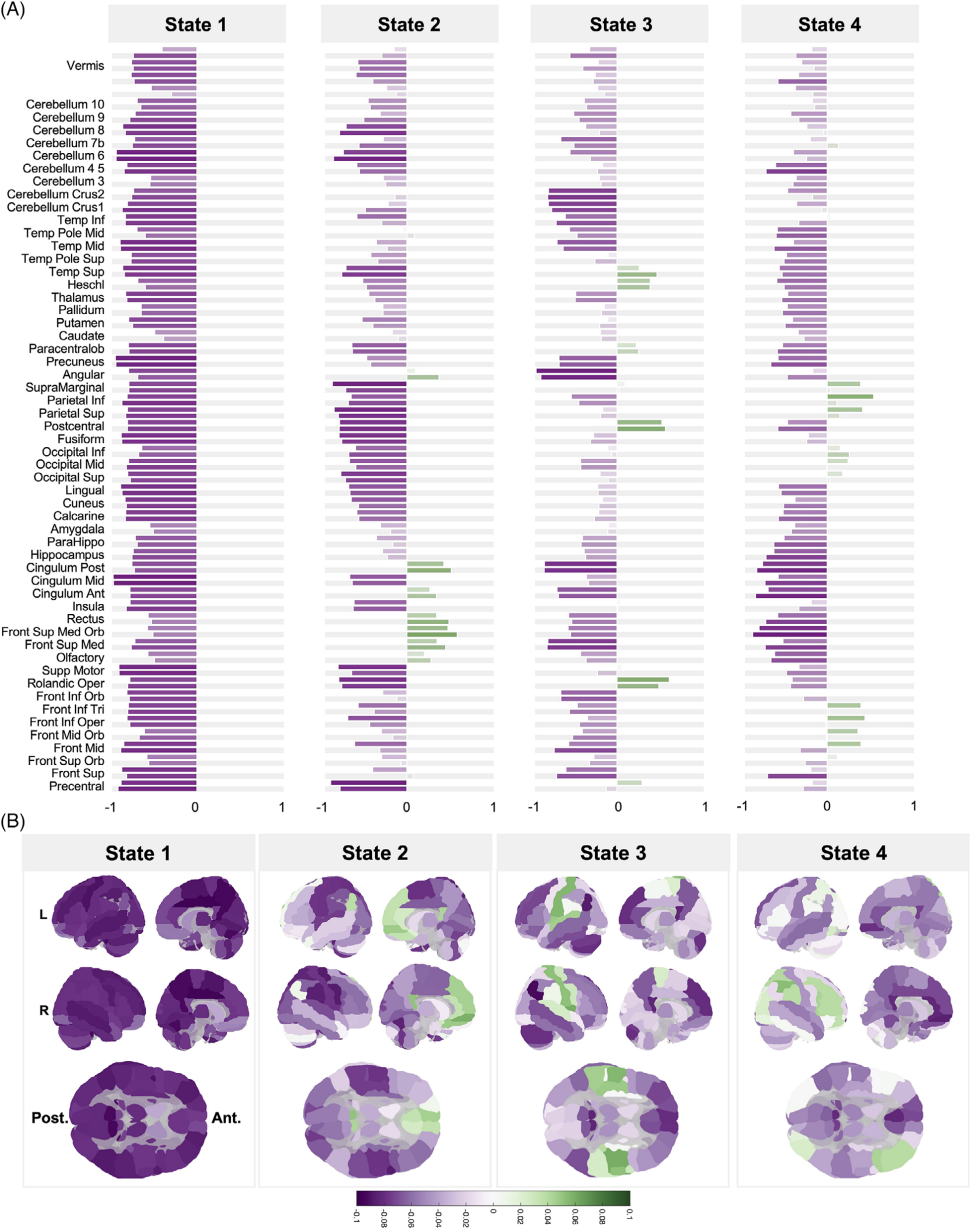
LEiDA‐derived brain states. (A) LEiDA vectors of all 116 AAL regions for each of the four brain states from k‐means clustering of the leading eigenvector of coherence matrices from all volumes across all participants. Top eight regions are vermis, below which each label refers to a pair of regions (right = white bar above, left = gray bar below). The leading eigenvectors had positive and negative signs dividing regions into two communities (green and purple). Darkness/length of bar represents the contribution of each region to the community. (B) Representation of the states on brain surfaces. Top row shows left side of the brain (L), lateral then medial aspect. Second row shows right side (R). Bottom row shows axial slice. Ant., anterior; LEiDA, Leading Eigenvector Dynamics Analysis; Post., posterior.

We also took a state‐based approach to further explore how AD affects global brain dynamics. Combining the resting‐state fMRI data from all participants, four brain states were defined using the LEiDA approach^32^ (Figure 1) (see Methods for details). To reduce dimensionality, this method only considers the leading eigenvectors of each phase coherence matrix. The leading eigenvectors are then clustered into four states using *k*‐means. Every fMRI volume from every participant was assigned to one of the four states, producing a state‐based time series per participant. State 1 shows widespread phase coherence between regions, as is routinely found when using the LEiDA approach, and is referred to as the ‘global coherence state’ (Figure 4).^32^, ^34^, ^66^, ^67^ State 2 consists of a phase coherence pattern where regions within the default mode network, including the superior frontal regions, the anterior and posterior cingulate, and the angular gyri have a phase in common with each other but distinct from the rest of the brain, representing a community. In State 3, there exists a community of sensorimotor and auditory regions, including the superior temporal regions, Heschl's gyri (primary auditory cortex), rolandic operculum, and primary sensorimotor cortices (pre‐ and post‐central gyri). Finally, State 4 shows coherence between frontoparietal network regions; the supramarginal gyrus, inferior and superior parietal lobe, inferior frontal gyrus, and middle orbitofrontal cortex, with the pattern of separation from the rest of the brain particularly clear for the right sided regions.

### 3.5 AD participants spend less time in the global coherence state and demonstrate less stable state dynamics

**FIGURE 5:**
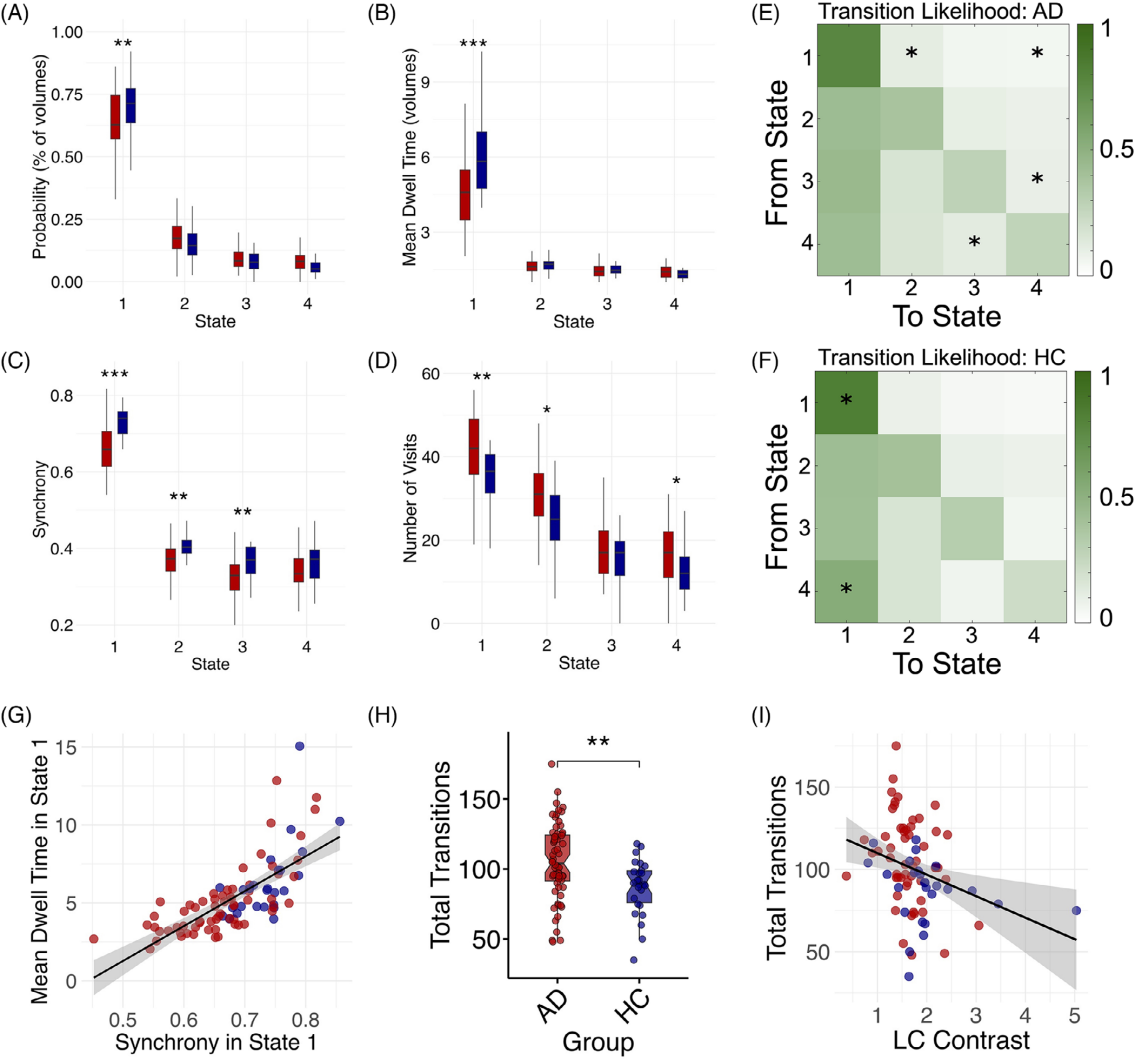
Brain state dynamics. Box plots comparing groups and states for: (A) probability (% of all volumes spent in this state); (B) mean dwell time (per separate visit, in volumes); (C) synchrony; and (D) number of visits by state. Significance indicators are the result of ANOVA applied to linear mixed effects models. (E/F) Transition matrices for AD and HC, respectively, including ‘transitions’ from state x to state x (i.e., consecutive volumes in the same state). Each row adds up to 1. * indicates significantly greater number of this transition in this group on *t*‐testing (FDR corrected). (G) Scatter plot showing relationship between State 1 synchrony and mean dwell time. (H) Box plot comparing HC and AD on the total number of transitions, significance indicator is the result of *t*‐test. (I) Scatter plot showing significant relationships between LC contrast and the total number of transitions. For illustrative purposes, linear best fit was plotted through raw data from all participants, however, note that statistical results reported in text include group as covariate. Shaded error bars show standard error of the mean. *p < 0.05; **p < 0.01; ***p < 0.001. AD, Alzheimer's disease (red); ANOVA, analysis of variance; FDR, false discovery rate; HC, healthy control (blue); LC = locus coeruleus.

Considering the distribution of state probabilities, defined as the fraction of fMRI volumes a participant spent in each state, there was a significant group:state interaction [F(3,360) = 6.35, *p* < 0.001]. The probability for State 1, the global coherence state, was 9.14% lower for AD than HC [*b *= −0.06, SE = 0.02, *t*(368) = −3.68, *p* < 0.01] (Figure 5A). For mean dwell time, the average number of continuous volumes spent per visit, there was also a significant group:state interaction [F(3,358) = 5.98, *p* < 0.001]; AD spent 20.78% less time in State 1 per visit than HC [*b *= −1.31, SE = 0.28, *t*(366) = −4.73, *p* < 0.001] (Figure 5B).

For each participant, synchrony was calculated for fMRI volumes assigned to each separate state. Synchrony was significantly and considerably higher for volumes assigned to State 1 compared to States 2–4 in both groups (Figure 5C), and there was a significant group:state interaction [F(3, 268) = 5.28, *p* = 0.001], with synchrony significantly lower for AD than HC for volumes in State 1 [*b *= −0.07, SE = 0.01, *t*(225) = −5.73, *p* < 0.001], 2 [*b *= −0.04, SE = 0.01, *t*(225) = −2.88, *p* < 0.01], and 3 [*b *= −0.04, SE = 0.01, *t*(230) = −2.87, *p* < 0.01]. Participants’ overall synchrony and State 1 probability were highly positively correlated [*b *= 1.23, SE = 0.08, *t*(84) = 14.54, *p* < 0.001]. Greater synchrony during State 1 correlated with longer mean dwell time [*b *= 0.02, SE = 0, *t*(84) = 8.53, *p* < 0.001] (Figure 5G).

AD participants made more separate visits to each of the four states, significantly so for State 1 [*b *= 6.09, SE = 1.89, *t*(220) = 3.22, *p* < 0.01], 2 [*b *= 4.56, SE = 1.89, *t*(220) = 2.41, *p* = 0.02], and 4 [*b *= 4.56, SE = 1.89, *t*(220) = 2.41, *p* = 0.02] (Figure 5D), and there was no group:state interaction. When in State 1, AD were more likely than HC to transition to State 2 [*p* = 0.04] and to State 4 [*p* = 0.03] (Figure 5E). When in State 1, HC were significantly more likely than AD to remain in that state rather than transition to an alternative state [*p* = 0.02]. Also, when in State 4, HC were more likely than AD to move into State 1 [*p* = 0.02] (Figure 5F). AD made 17.23% more transitions overall [*b *= 0.69, SE = 0.22, *t*(86) = 3.10, *p* < 0.01]. Greater LC contrast related to fewer transitions across all participants [*b *= −0.22, SE = 0.11, *t*(74) = −2.05, *p* = 0.04], and there was no group interaction [*p* = 0.40] (Figure 5I).

### 3.6 Pupil‐linked arousal tracks synchrony and the global coherence state

**FIGURE 6:**
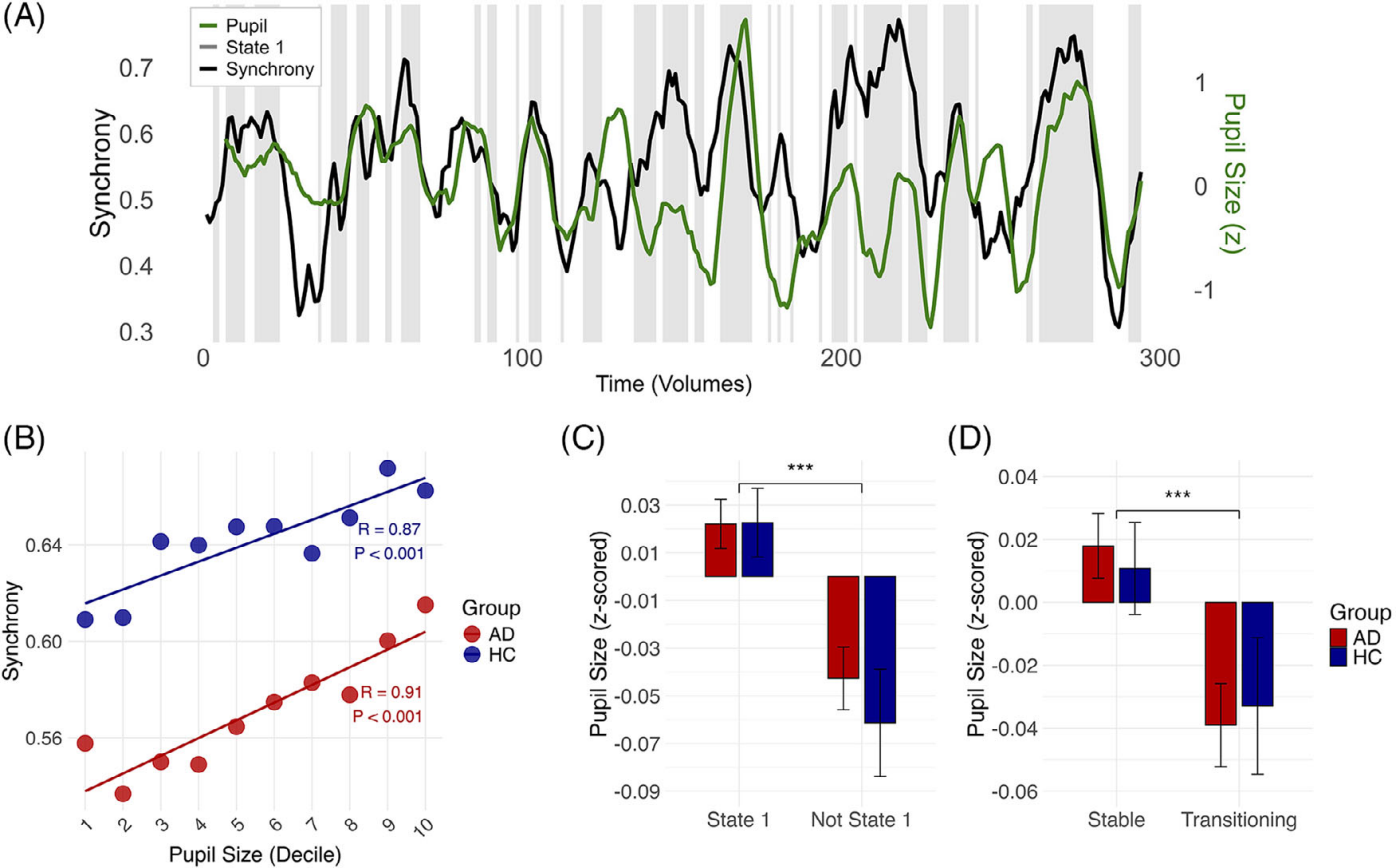
Pupil‐linked arousal and brain dynamics. (A) Example participant's synchrony (black) and pupil (green) timecourses (smoothed for visualisation purposes). Time in State 1 denoted by gray areas, demonstrating pupil size is usually increased during State 1. (B) Pupil decile strongly correlates with synchrony when averaged across subjects. R and *p*‐values from Pearson's correlation. (C) Pupil size on average is greater when in State 1, versus when not in State 1, and (D) in volumes that do not proceed a transition to another state (stable), versus volumes that do (transitioning), with no group:condition interaction for either. Error bars show standard error of the mean. Significance indicators are the result of mixed effects models accounting for participant as repeated measure. ***p < 0.001. AD, Alzheimer's disease (red); HC, healthy control (blue).

Having established a link between the structural integrity of the LC and synchrony, we tested the hypothesis that synchrony would increase and decrease in line with fluctuations in arousal. Measuring pupil size during the resting‐state fMRI sequence allowed us to do this by providing a measure of arousal simultaneously with cortical brain dynamics. The correlation coefficients (Pearson's R) between participants’ pupil size and synchrony over time were significantly different from zero [*t*(78) = 5.11, *p* < 0.001], indicating an overall positive correlation between pupil size and synchrony across the participants. This was also true of the HC and AD groups analysed separately, and there was no significant group difference in pupil‐synchrony correlations [*p* = 0.67]. To further confirm this, fMRI volumes were then binned into deciles based on the pupil size and the synchrony was averaged for all volumes in each bin at the participant and then group level. Pupil bin and synchrony were highly correlated (Pearson's R) for both groups [HC: R = 0.87; AD: R = 0.91] (Figure 6B).

We then explored the relationship between arousal and cortical dynamics characterised in terms of LEiDA states. Linear mixed effects models on all volumes, with participant as a repeated measures term, revealed pupil size was significantly greater when in State 1 versus when not in State 1 [*b *= 0.06, SE = 0.02, *t*(21870) = 3.83, *p* < 0.001] (Figure 6C). To test the relationship between arousal and the stability of state occupancy, volumes were also divided into those which preceded a change in state (transitioning) and those that did not (stable). Pupil size was significantly smaller in the transitioning condition [*b *= −0.06, SE = 0.02, *t*(21800) = −3.36, *p* < 0.001] (Figure 6D). There was no significant group:condition interaction, indicating that the relationship between condition and pupil size did not differ between groups for either model. LC integrity did not relate to z‐scored pupil size during any of the four states, or when stable or transitioning, for all subjects or for either group separately [all *p* > 0.05]. These results show that higher pupil‐linked arousal relates to periods of high synchrony and periods in the global coherence state, and to stability in terms of state dynamics, that is, a propensity to remain in the current state.

## 4 DISCUSSION

Synchronised cortical activity is required for multiple cognitive functions.^11^, ^68^ Using fMRI in AD, we showed reductions in synchrony that relate to cognition. The reductions in synchrony in AD were of a greater magnitude than that of the other MRI measures, including global gray matter volume and white matter integrity. Arousal state was strongly associated with these abnormal cortical dynamics, as demonstrated through the correlations between pupil size and instantaneous global synchrony. In addition, LC integrity, measured using contrast on neuromelanin MRI, was reduced in AD and related to cognition.^21^ We propose arousal dysfunction in AD contributes to a failure to maintain synchronous, yet flexible, patterns of cortical activity, in part the result of disruption to ascending noradrenergic pathways from the LC.

Synchrony is thought to support information exchange across spatially distributed neuronal populations.^68^, ^69^, ^70^ We tested whether large‐scale synchrony is disrupted in AD by measuring the phase coherence of fluctuations in fMRI BOLD across brain regions. This coherence approach allowed the estimation of an instantaneous measure of global connectivity whilst a dynamic analysis could be linked to arousal fluctuations.^32^ Previous AD work has predominantly used EEG‐derived synchrony.^12^, ^14^ Reduced synchrony in AD during resting‐state relates to overall cognitive dysfunction,^71^, ^72^ with a similar association seen during cognitive tasks.^73^ For example, lower coherence in event‐related EEG oscillations across frequency bands during an attentionally demanding oddball task has been reported.^74^ A small number of AD fMRI studies have shown reductions in synchrony, in keeping with our results.^4^, ^30^

LEiDA studies in healthy and clinical populations have shown that less time spent in the “global coherence state” (State 1), that is, a failure to maintain synchronous activity, is typically seen in the context of cognitive dysfunction.^34^, ^59^, ^66^, ^75^ We report reduced time spent in State 1 in AD, consistent with other studies and aligning with our hypothesis,^5^, ^33^, ^34^ although one recent LEiDA study found an *increased* time spent in this state.^33^ Metastability (the standard deviation of synchrony over time) provides a further measure of how the brain forms complex, time‐varying connectivity patterns.^2^, ^76^ Here, metastability was significantly reduced in AD, in keeping with previous work.^4^, ^5^, ^30^ This may be relevant to cognitive impairments seen in AD. In a large study of healthy individuals, resting‐state metastability predicted performance across multiple in‐scanner tasks, suggesting it reflects how readily the brain can reorganise in response to task demands.^77^ In traumatic brain injury, we previously showed metastability to correlate positively with performance in memory, attention, and cognitive flexibility tasks.^31^ Similar tasks could be applied to test these relationships in AD.

**FIGURE 7:**
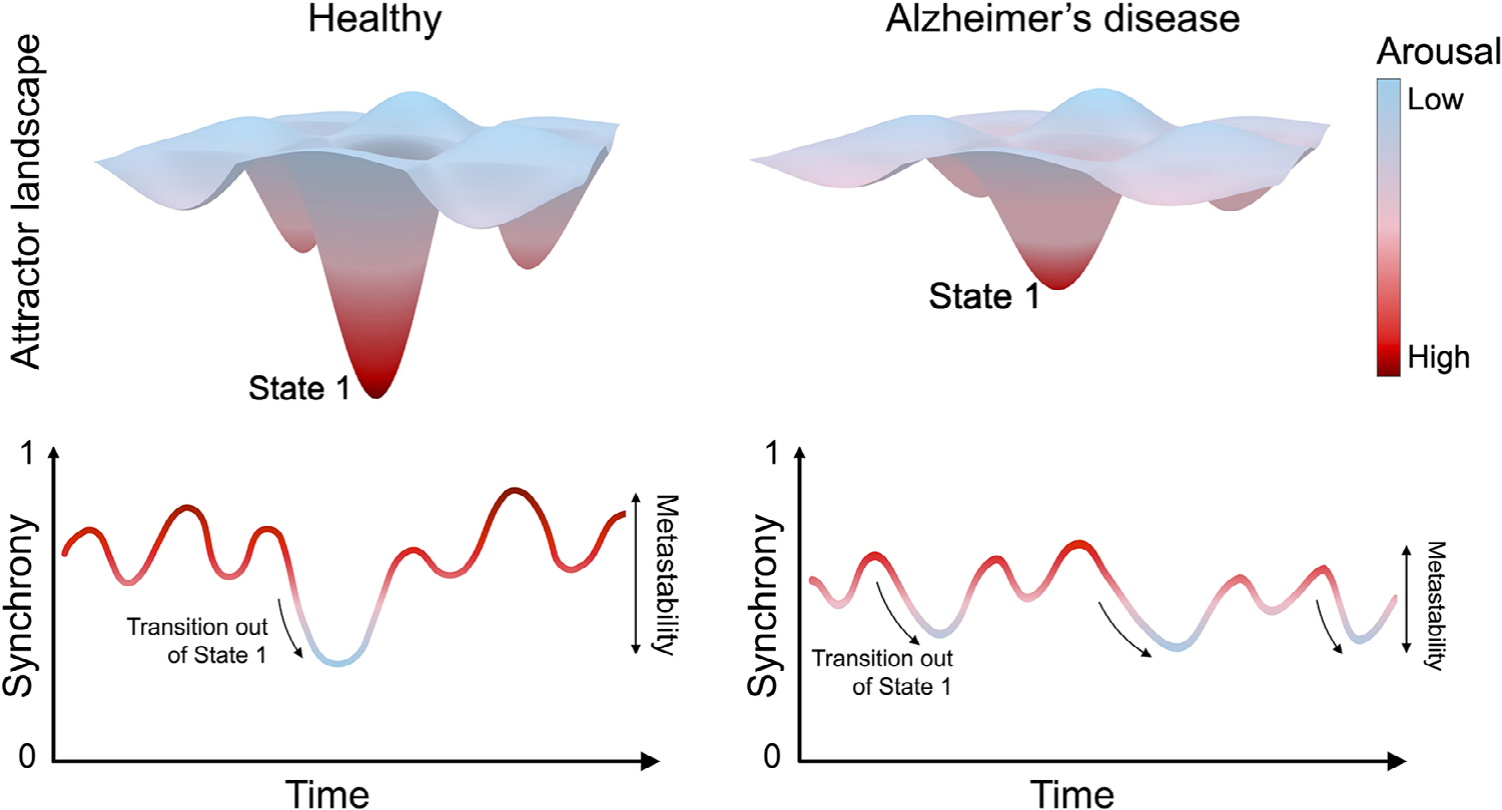
Schematic representation of a model describing how arousal and disease state may affect the brain's attractor landscape. Top: Attractor landscapes – theoretical constructs describing the tendency for the brain to transition into and out of different states – in the healthy (left) and AD (right) brain. State 1 represents the LEiDA “global coherence state”—the predominant state in which the brain resides. Bottom: An illustrative example timecourse of global synchrony in the healthy (left) and AD (right) brain. The healthy brain exhibits a higher average synchrony but also a greater variability, that is, metastability. Arousal level correlates with synchrony, so when arousal falls (bluer colors), synchrony does too and the brain is more likely to transition out of the predominant attractor (State 1). According to this model, in AD, attenuated arousal tone means that State 1 is shallower and therefore less persistently occupied, leading to more frequent transitions to other states (indicated with curved, downwards arrows). The colors represent arousal level. AD, Alzheimer's disease; LEiDA, Leading Eigenvector Dynamics Analysis.

Analysis of the transition behaviour between LEiDA states allowed further exploration of AD‐related alterations in cortical dynamics. The tendency for subjects to remain in, or transition from, states can be conceptualised within an “attractor landscape”.^2^ We found AD patients visit State 1 more often but spent less time there overall, accompanied by a greater number of overall state transitions. This could be explained by State 1 having a shallower attractor depth in AD, that is, being less stable. A greater number of transitions might seem inconsistent with lower metastability in AD. However, synchrony during State 1 was higher in controls than in AD, allowing for greater metastable fluctuations when in this state, and requiring larger changes in synchrony to transition from it (Figure 7).^78^ Accordingly, the higher the synchrony during State 1, the longer an individual dwelled there, indicating a deeper attractor state. When controls left State 1, they briefly visited partially synchronised, weakly stable states before returning. This reflects a metastable system in the healthy brain, where dominant attractor states are both frequently occurring and relatively long‐lived.^2^, ^61^ In contrast, the pattern in AD of frequent transitions yet lower metastability may reflect reduced stability of the global coherence state, rather than greater dynamic variability. This corroborates previous work showing major attractor states are shallower and less easily maintained in AD, resulting in unstable state dynamics.^79^, ^80^

We show that cortical dynamics are strongly related to arousal in both health and AD. Arousal is an autonomic process affecting the whole body.^81^ Within the brain, increased arousal is marked by the release of neurotransmitters from ascending projections to the cortex, including noradrenaline from the LC.^20^ Cortical dynamics measured using MEG and fMRI are known to correlate strongly with physiological markers of arousal state, including pupil size, during rest.^6^, ^25^ For example, both spontaneous phasic arousal events (K‐complexes during sleep) and those induced by a reaction‐time task produce tightly coupled changes in autonomic physiology and the global BOLD signal.^81^ We have extended this work to demonstrate that strong correlations between pupil size (a proxy for LC activity) and cortical dynamics are preserved in AD.^8^

AD neuropathology affects key brainstem nuclei involved in the control of arousal state, notably the LC, early in the disease course.^16^ Accordingly, we replicated previous findings of reduced LC integrity in AD.^21^ We have previously shown a relationship between reduced LC integrity and cortical slowing, a putative measure of arousal state.^8^ Here, reduced LC integrity was associated with reduced cortical synchrony, providing further evidence that arousal system dysfunction relates to the changes in cortical dynamics seen in AD. Unlike this specific marker of damage to the arousal system, other measures of neurodegeneration—gray matter volume and mean FA of white matter tracts—did not explain synchrony changes. However, the LC is unlikely to be unique as a region where the degree of neurodegeneration relates to global measures of synchrony or cognition, and work systematically exploring other cortical and sub‐cortical regions would be an interesting future direction.

Regarding the attractor landscape, our results support that arousal acts to increase the depth of attractor states alongside increasing their neuronal synchrony (Figure 7).^1^, ^15^ As arousal drops, the brain transiently transitions into segregated states.^78^, ^82^, ^83^, ^84^ This was seen here, with larger pupil size during the high‐synchrony state and at times of state stability. This was further supported by a negative relationship between LC integrity and the number of state transitions. As such, a healthier arousal system stabilises state dynamics, allowing only infrequent visitations to segregated states. It is plausible that attenuated arousal function in AD is insufficient to produce and maintain synchronised neuronal activity. This could result in an inability to sustain attention, perform working memory tasks, and other executive functions.^1^, ^70^

Our findings can also be interpreted through the framework of criticality.^85^ The brain can be viewed as a complex dynamical system operating at the boundary between system‐wide order and disorder (i.e., at the critical point). Using voltage imaging in mice, we previously showed that when waking from anaesthesia, scale‐invariant spatiotemporal patterns of neuronal activity emerge, consistent with the awake cortex operating at the critical point.^86^ This scale‐invariant (fractal) organisation is a feature of critical dynamics optimised for information exchange.^87^ Furthermore, at this point, systems of oscillators exhibit variability in phase coherence, that is, metastability.^85^, ^88^ In situations of reduced synchrony such as those with moderate‐severe AD included here, cortical dynamics are pushed away from a metastable “sweet‐spot”. This finding adds to mounting evidence that AD causes a deviation from criticality.^89^, ^90^ We and others have previously argued that arousal is a key modulator of such dynamics, balancing the system at the critical point,^91^, ^92^ and supporting metastability.^77^, ^78^, ^93^ Arousal dysfunction may therefore explain the changes in criticality seen in AD.^91^

Our findings highlight the potential of arousal‐modulating treatments to restore synchronised brain dynamics and improve cognitive symptoms in AD.^94^, ^95^ Catecholaminergic drugs induce functional connectivity changes that relate to improvement in cognition in health^96^, ^97^, ^98^ and traumatic brain injury,^99^ whilst in Parkinson's disease, dopaminergic therapy increases synchrony and metastability.^100^ In AD, the benefits of cholinergic medications for cognitive symptoms^101^ may be partially mediated by their action on arousal.^19^ However, neuroimaging studies of the actions of arousal‐modulating drugs on cortical activity in AD are very limited.^102^ Synchrony, as a global measure which we have shown relates strongly to arousal, could be useful to index the effects of drugs and other therapies.^103^, ^104^

This study had some limitations. First, pupil size represents only a proxy of activity in arousal nuclei, including, but not limited to, the LC.^105^ A more direct approach would be to delineate BOLD activity within arousal‐modulating regions (e.g. LC, substantia nigra, basal forebrain) using ultra‐high‐field fMRI, to understand the specific contribution of each neuromodulatory system to brain dynamics.^106^ Second, more extensive neuropsychological testing could provide a more granular cognitive profile and differentiate between control participants performing near ceiling. Such measures may be more sensitive to inter‐subject differences or map closer to brain dynamics. Nevertheless, the ACE is a widely used, clinically validated tool with domain specific effects in this study. In addition, more extensive testing would likely have been impractical to perform given the degree of impairment of many of the AD participants. Third, our methodology precluded the assessment of how metastability fluctuates with arousal state within individuals. This is because metastability, here defined as the standard deviation of the KOP, inherently requires averaging over an extended time window. Fourth, the lack of amyloid biomarkers for some AD participants limits our ability to ascertain whether the effects of arousal dysfunction demonstrated are specific to AD pathology. However, they remain relevant for understanding the effects of neurodegeneration of the LC that is seen in a range of disorders.^107^ Fifth, whilst we controlled for confounding factors such as disease stage measured by length of symptoms, and imaging measures of global atrophy, there is likely a degree of collinearity between co‐variates, and using them for cross‐sectional adjustment cannot confirm mechanistic independence.

The brain is a complex dynamical system, tuned to support information processing by the tight control of cortical synchrony.^88^ AD perturbs the system, leading to cognitive failures. We showed that synchrony, measured using phase coherence of fMRI BOLD, was significantly reduced in AD and related to cognitive dysfunction. Variability in synchrony is also indicative of healthy brain function and was also reduced in AD. Synchrony reflects arousal function as demonstrated through correlations with pupil size and LC integrity. Arousal function is attenuated in AD due to pathology in key structures. This may lead to reduced synchrony and flatten the attractor landscape, destabilising cortical dynamics and producing cognitive impairment.

## CONFLICT OF INTEREST STATEMENT

PM is lead for an NIHR‐funded trial with drug/placebo provided by Takeda Pharmaceuticals and sits on the Data Monitoring Committee for a trial carried out by Johnson and Johnson. He is vice chair of the Alzheimer's Society Research Strategy Council, and NIHR Specialty Lead for Dementia and Neurodegeneration, Research Delivery Network. He is also an independent member of a data monitoring committee. All other authors have nothing to disclose. Author disclosures are available in the Supporting Information.

## CONSENT STATEMENT

The Minder and PCNorAD studies were approved by the London‐Surrey Borders Research Ethics Committee (19/LO/0102) and London‐Central Research Ethics Committee (18/LO/0249) respectively. All participants with capacity to consent provided written informed consent for participation and for their data to be included. Those without capacity were enrolled in accordance with the Mental Capacity Act (2005) on recommendation of an assigned consultee.

## Supporting information



Supporting Information

Supporting Information

## Data Availability

Data and code including pupil pre‐processing pipeline are available upon reasonable request.
