# Supplementary material for: Cortical synchrony is reduced in Alzheimer's disease and relates to arousal state
Source: Alzheimers Dement. 2026 Jun 11;22(6):e71547. doi: 10.1002/alz.71547 (PMC13254816; doi:10.1002/alz.71547)
Supplement: Supplementary file 1 — Supporting Information [file ALZ-22-e71547-s001.docx]

**Supplementary Material**

### **Supplementary Methods**

**DTI analysis**

Motion and eddy current artefacts were first corrected, and the corresponding b‐vectors were rotated. Skull stripping was performed using BET. Tensor fitting employed a weighted least squares method to produce fractional anisotropy (FA) maps, which were subsequently registered to MNI space using the FIMRIB 1mm FA atlas in Tract-Based Spatial Statistics.^1^ After skeletonisation at an FA threshold of 0.2 to retain only the central portions of white matter tracts, the mean FA across the entire skeleton was calculated for each participant as an overall measure of white matter integrity.

**fMRI data cleaning**

Functional imaging pre-processing was performed using FEAT, in FSL.^2^ This involved realignment of EPI images and spatial smoothing using a 6mm full-width at half-maximum Gaussian kernel. fMRI data were registered to individuals’ T1 images with FLIRT.^3^ FMRIB’s Automated Segmentation Tool^4^ was used to produce segmentations of the cerebrospinal fluid and white matter from the T1, and then these masks were applied to the fMRI data to regress out any signal from these sources.

Cleaning of movement and other sources of noise was then conducted. In terms of correcting for movement artefact, first, framewise displacement (FD), a measure of head movement from one frame to the next,^5^ was obtained for each volume using the FSL motion outliers tool. Although previous work in AD has not chosen to exclude participants based on mean FD,^6–8^ we took a more stringent approach and excluded scan visits with a mean FD >0.55.^9^ Of the acquisitions selected for pre-processing, 18/82 (22%) AD and 1/27 (4%) HC participants were excluded on FD. In those kept for analysis, there was no group difference in FD [P>0.05], and the final group-level mean FD [HC=0.29; AD=0.31] was comparable to other work.^8^ MCFLIRT^3^ was used to estimate motion to 6 degrees of freedom. The 6 motion estimates and their 24 temporal derivatives were also applied as nuisance regressors to derive motion-corrected images. Also, pre-processed fMRI data were decomposed into distinct spatial and temporal components and each component was assessed as to whether it was likely to be noise, using ICA-AROMA.^10^ All components were then also visually inspected using their spatial maps, time series, and power spectra, and those considered to be noise as per published guidance^11^ were removed, with the rater blinded to the outcome of the automated assessment.

### **Supplementary Results**


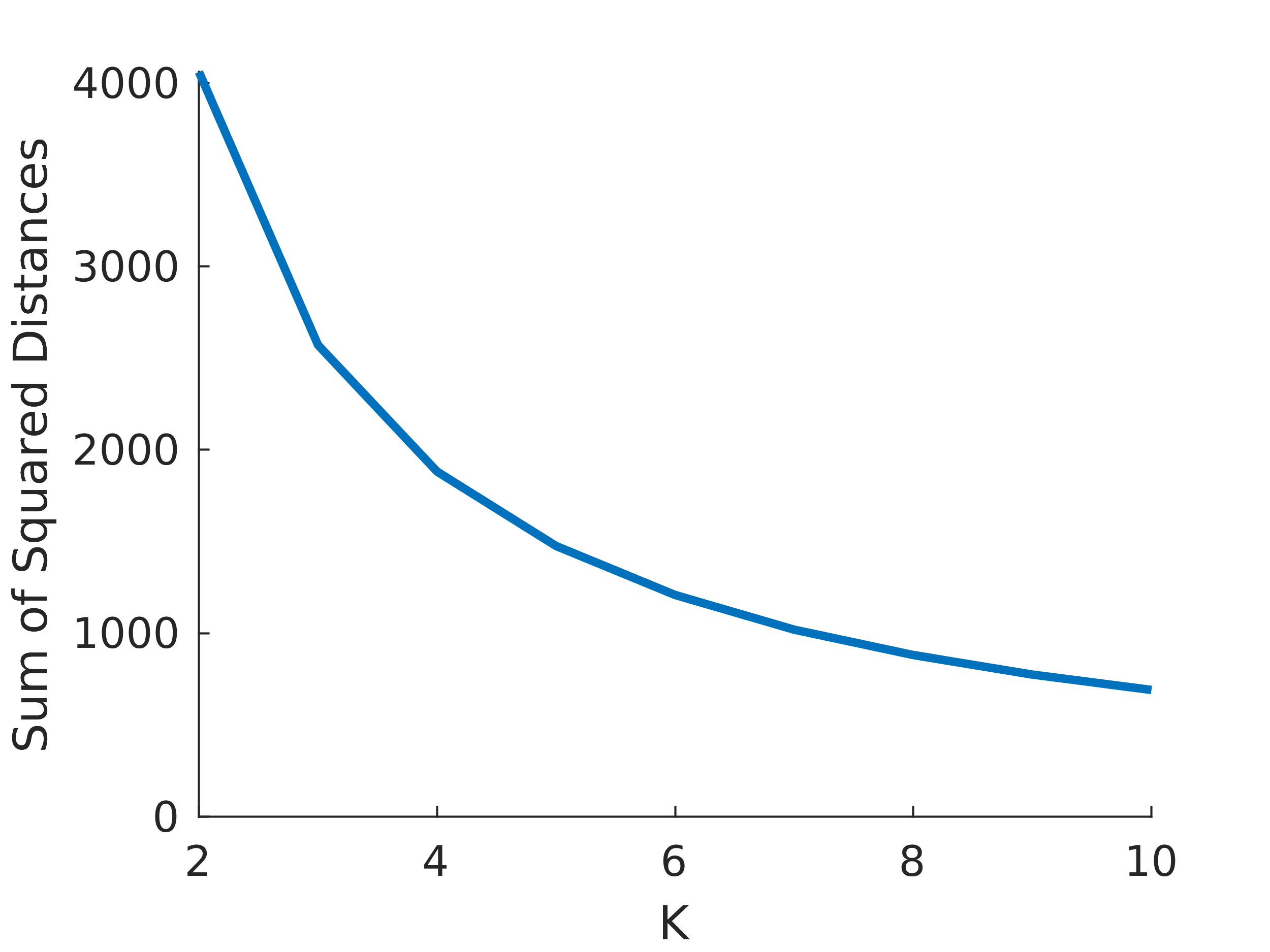

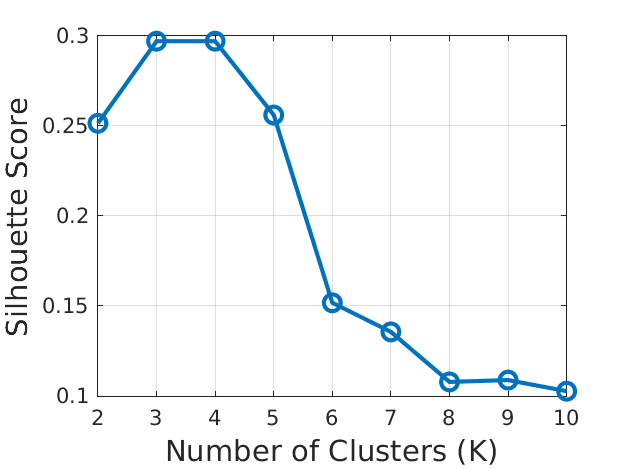


**A)**

**B)**

***Supplementary Figure 1.* K*-means clustering of LEiDA states.*** ***A)*** *Elbow plot showing how the sum of squared distances decreases with increasing number of clusters (*k*). The steeper drop from* k*=2 to* k*=4 suggests that adding more clusters beyond that point yields diminishing returns in terms of within-cluster variance reduction.* ***B)*** *Optimal cluster solution, from the range of* k*=2-10, was 4 based on the maximum Silhouette score. LEiDA = Leading Eigenvector Dynamics Analysis.*


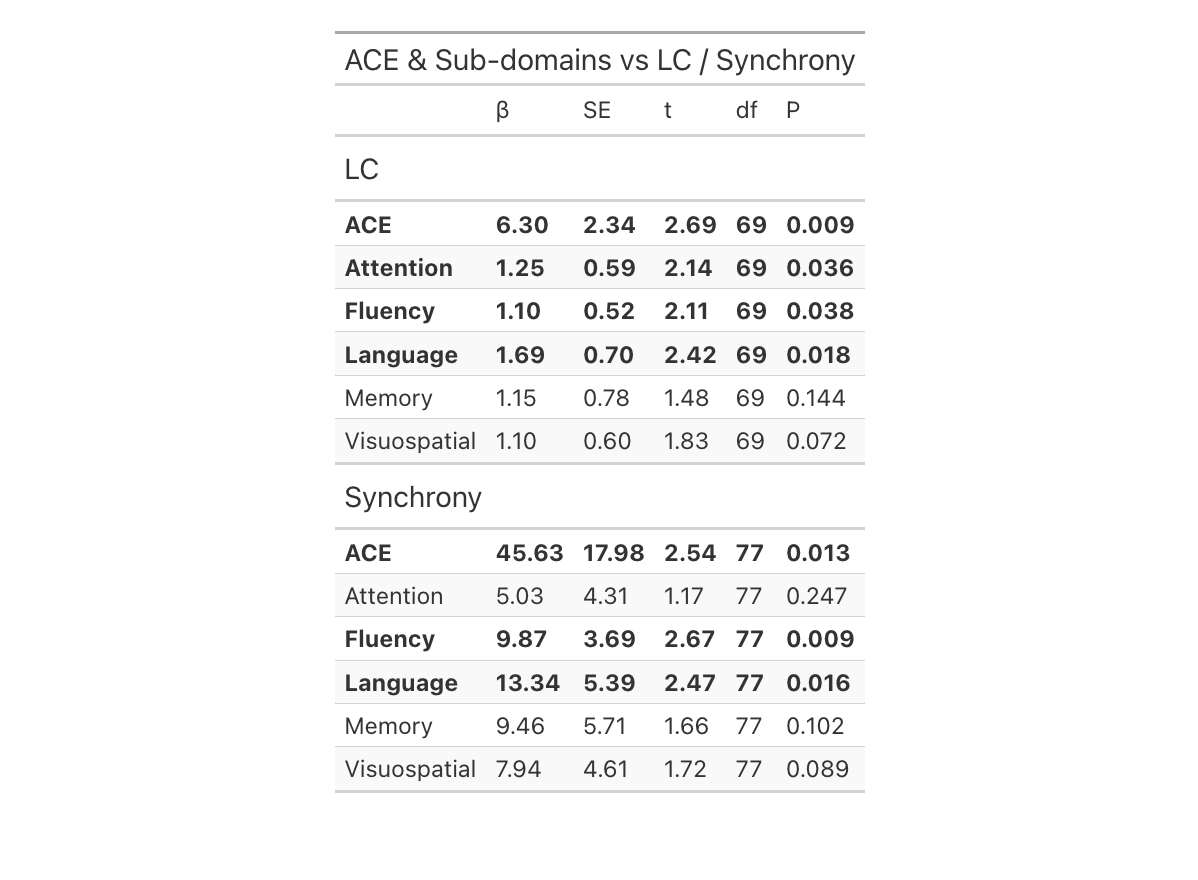


***Supplementary Table 1. LC contrast and synchrony in relation to ACE scores.*** *Results from linear models including all subjects accounting for group and other co-variates, not corrected for multiple comparisons. LC = locus coeruleus, ACE = Addenbrooke’s Cognitive Examination – III total score.*

**References**

1. Smith SM, Jenkinson M, Johansen-Berg H, Rueckert D, Nichols TE, Mackay CE, et al. Tract-based spatial statistics: Voxelwise analysis of multi-subject diffusion data. Neuroimage. 2006;31(4):1487–505.

2. Smith SM, Jenkinson M, Woolrich MW, Beckmann CF, Behrens TEJ, Johansen-Berg H, et al. Advances in functional and structural MR image analysis and implementation as FSL. Neuroimage. 2004;23(SUPPL. 1):208–19.

3. Jenkinson M, Bannister P, Brady M, Smith S. Improved Optimization for the Robust and Accurate Linear Registration and Motion Correction of Brain Images. Neuroimage. 2002;17(2):825–41.

4. Zhang Y, Brady M, Smith S. Segmentation of brain MR images through a hidden Markov random field model and the expectation-maximization algorithm. IEEE Trans Med Imaging. 2001;20(1):45–57.

5. Power JD, Barnes KA, Snyder AZ, Schlaggar BL, Peterson SE. Spurious but systematic correlations in functional connectivity MRI networks arise from subject motion. Neuroimage. 2012;59(3):2142–54. Available from: https://www.ncbi.nlm.nih.gov/pmc/articles/PMC3624763/pdf/nihms412728.pdf

6. Mascali D, DiNuzzo M, Serra L, Mangia S, Maraviglia B, Bozzali M, et al. Disruption of Semantic Network in Mild Alzheimer’s Disease Revealed by Resting-State fMRI. Neuroscience. 2018;371(2018):38–48. Available from: https://doi.org/10.1016/j.neuroscience.2017.11.030

7. Zhao S, Rangaprakash D, Venkataraman A, Liang P, Deshpande G. Investigating focal connectivity deficits in Alzheimer’s disease using directional brain networks derived from resting-state fMRI. Front Aging Neurosci. 2017;9(JUL):1–12.

8. Scherr M, Utz L, Tahmasian M, Pasquini L, Grothe MJ, Rauschecker JP, et al. Effective connectivity in the default mode network is distinctively disrupted in Alzheimer’s disease—A simultaneous resting-state FDG-PET/fMRI study. Hum Brain Mapp. 2021;42(13):4134–43.

9. Satterthwaite TD, Wolf DH, Loughead J, Ruparel K, Elliott MA, Hakonarson H, et al. Impact of in-scanner head motion on multiple measures of functional connectivity: Relevance for studies of neurodevelopment in youth. Neuroimage. 2012;60(1):623–32. Available from: http://dx.doi.org/10.1016/j.neuroimage.2011.12.063

10. Pruim RHR, Mennes M, van Rooij D, Llera A, Buitelaar JK, Beckmann CF. ICA-AROMA: A robust ICA-based strategy for removing motion artifacts from fMRI data. Neuroimage. 2015;112:267–77. Available from: http://dx.doi.org/10.1016/j.neuroimage.2015.02.064

11. Griffanti L, Douaud G, Bijsterbosch J, Evangelisti S, Alfaro-Almagro F, Glasser MF, et al. Hand classification of fMRI ICA noise components. Neuroimage. 2017;154(December 2016):188–205.
